# Extraskeletal Osteosarcoma of the Thigh: An Autopsy Case Report

**DOI:** 10.1155/2009/186565

**Published:** 2009-06-02

**Authors:** Akihito Nagano, Takatoshi Ohno, Yutaka Nishimoto, Kazunari Yamada, Katsuji Shimizu

**Affiliations:** ^1^Department of Orthopaedic Surgery, Graduate School of Medicine, Gifu University, 1-1 Yanagido, Gifu 501-1194, Japan; ^2^Department of Surgical Nursing, Graduate School of Medicine, Gifu University, 1-1 Yanagido, Gifu 501-1194, Japan

## Abstract

We report a case of extraskeletal osteosarcoma (ESOS) and autopsy findings. A 35-year-old man presented with an ossified tumor in the right thigh and lung metastasis. The lung tumors continued to develop despite multiagent chemotherapy and caused death within 8 months. Autopsy revealed many secondary lesions in the lungs, especially in the left lung. Histopathologically, the primary tumor and one of the secondary tumors showed proliferation of spindle-shaped tumor cells focally forming lace-like osteoid material. Therefore, we made a definite diagnosis of ESOS.

## 1. Introduction

Extraskeletal osteosarcoma (ESOS) is a rare malignant mesenchymal neoplasm in soft tissues but not directly attached to the skeletal system. It accounts for less than 4% of all osteosarcomas and approximately 1.2% of all soft tissue sarcomas [1]. ESOS was first reported by Wilson [2], and fewer than 300 cases have been reported to date [3]. Here, we describe a case of ESOS in the thigh and autopsy findings.

## 2. Case Report

### 2.1. Background

A 35-year-old man presented with a swelling in his right thigh, which he had first noticed one month before. His medical history was unremarkable. On physical examination, a well-circumscribed, elastic-firm soft tissue mass was palpated. The mass was attached to deep fascia and was not mobile. Its maximum width was about 16 cm. The skin over the swelling was slightly reddish and showed no signs or either ulceration or necrosis, but was varicosed. Laboratory tests revealed slight increase in the white blood cell count (8800) and alkaline leukocyte phosphatase (ALP) (330), but no other abnormalities.

### 2.2. Radiographic Findings

**Figure 1:**
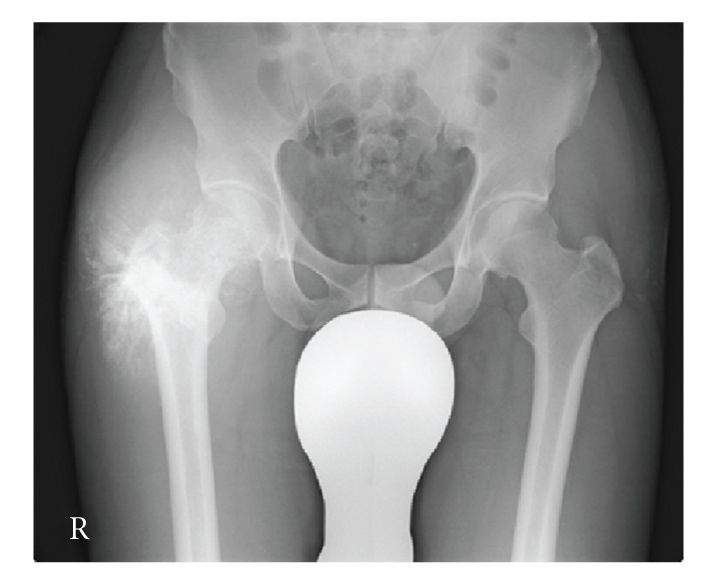
Plain radiography at first visit. Marked calcification was observed in the right thigh.

**Figure 2:**
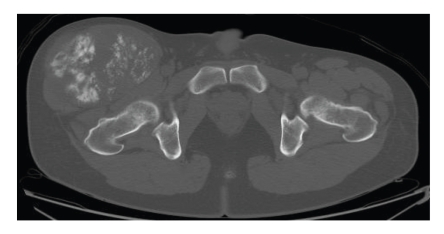
Computed tomography of thigh. The tumor was not in contact with the femur or its periosteum.

**Figure 3:**
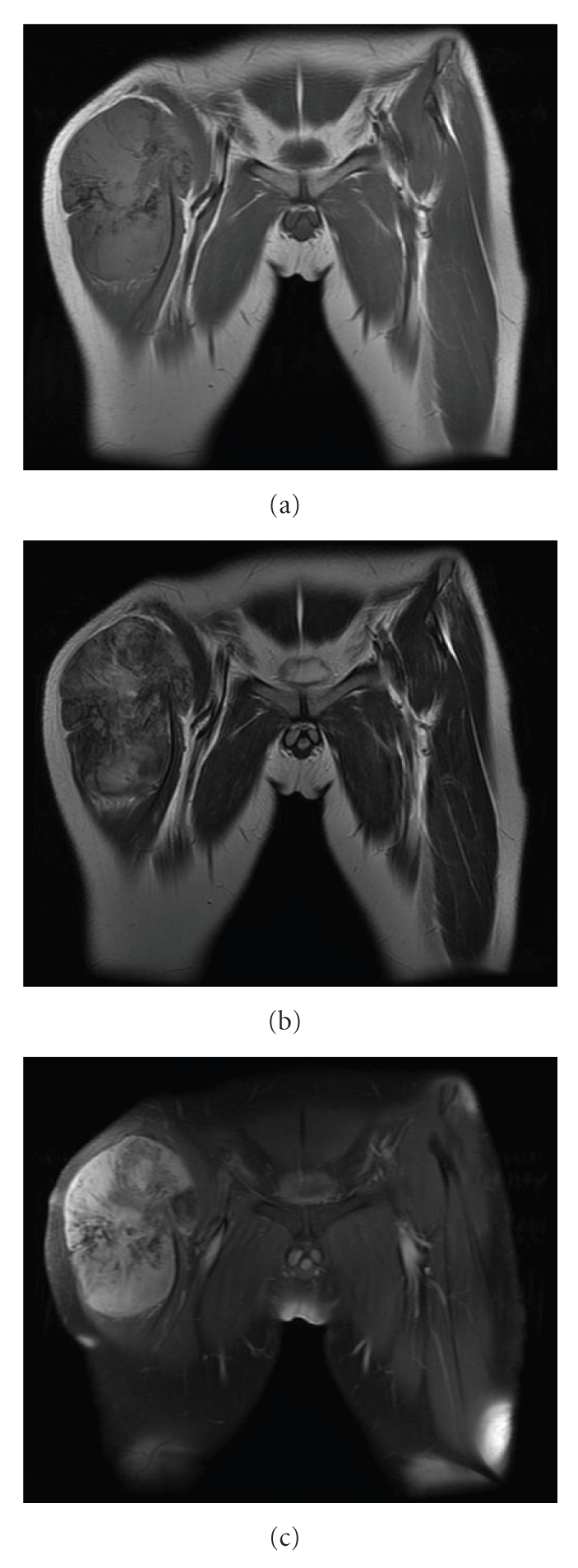
Magnetic resonance imaging of the tumor. (a) T1-weighed MRI. The mass showed mixed low and iso-intensity signals. (b) T2-weighed MRI. The mass showed mixed high and iso-intensity signals. (c) The tumor was enhanced remarkably by gadolinium.

**Figure 4:**
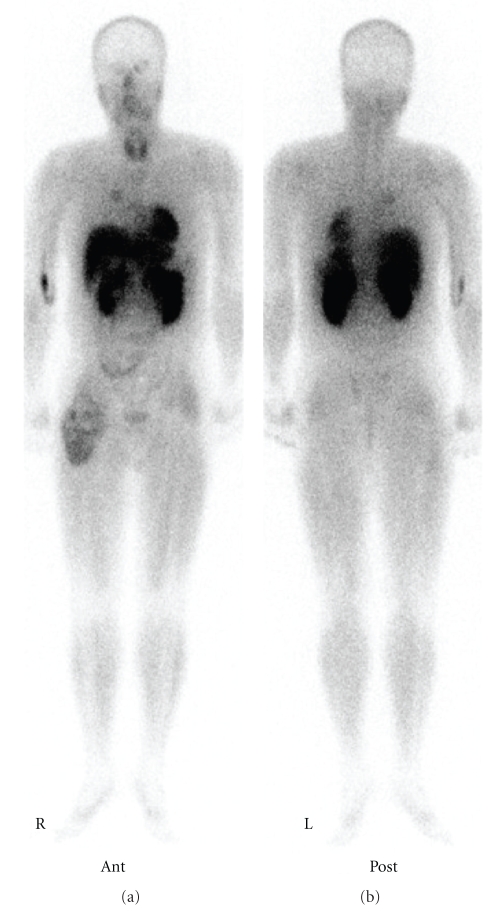
Gallium scintigraphy showed increased uptake in the primary tumor only.

Plain radiography of the right thigh showed a large soft tissue mass with marked calcification in a radial pattern (Figure 1). Computed tomography (CT) showed that many refined calcifications were scattered in the mass, and it was not in contact with the femur or pelvis (Figure 2). Magnetic resonance imaging (MRI) showed that the mass was well defined and located between the sartorius muscle and tensor fasciae latae muscle. The mass showed mixed low and iso-intensity on T1-weighted images and mixed high and iso-intensity on T2-weighted images. After intravenous administration of a gadolinium-based contrast material, the tumor was well enhanced (Figure 3). Calcifications present as signal voids on all sequences. No marked high intensity areas were observed on T2-weighted images. There were no obvious findings of hemorrhage or necrosis. Gallium scintigraphy showed marked heterogeneous accumulation especially in distal part of tumor (Figure 4). Therefore, it was considered that differential diagnosis contains myositis ossificans, synovial sarcoma, extraskeletal chondrosarcoma, extraskeletal osteosarcoma, malignant fibrous histiocytoma (MFH), and liposarcoma. 

**Figure 5:**
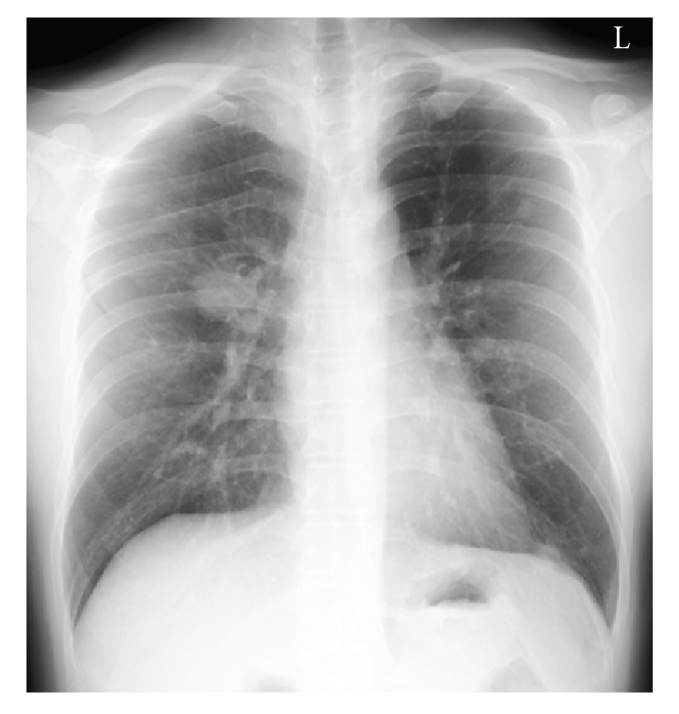
Plain radiography of lung. Multiple metastatic lesions in both lungs were found at first visit.

Plain chest radiography revealed 8 nodular densities in the left lung and 6 in the right, which were considered to be metastasis (Figure 5). The largest nodule was seen at right pulmonary hilum. The chest CT revealed multiple masses, irregular in size, with no pleural effusions and no mediastinal lymphnode hyperplasia. 

Regarding the findings described above, the biopsy site of the tumor in the right thigh was chosen to contain calcification and well-enhanced areas with gadolinium both of which were considered as signs of tumor aggressiveness. Indeed, the biopsy site chosen included the antero-lateral and distal sections of the tumor. The deep portion of the tumor was excised along with the peripheral portion so as to obtain both calcification and noncalcification areas.

An open biopsy specimen had malignant osteoid in lace-like formations of long or short spindle cells with osteogenesis and chondrogenesis. Hypercellularity and cellular pleomorphism were observed. The number of mitotic figures was seen about five mitosis/10 high-power fields (HPF). Immunohistochemically, the tumor showed reactivity with antibodies against vimentin, desmin, and CD68. The MIB-1 labeling index was 30–40%, and S-100, PAS, SMA, EMA, and keratin showed no reactivity. Thus, the tumor was diagnosed as ESOS.

### 2.3. Chemotherapy

Multiple metastases in both lungs were considered as inoperable. Moreover, from the aspect of pain control, there is little point in excising the primary tumor because the patient did not feel any pain. Therefore chemotherapy was primarily chosen as palliative treatment. The chemotherapy regimen referred to Kanazawa University protocol [4] was modified.

The doses of the antitumor agents were as follows: first and second cycles: cisplatin 460 mg, doxorubicin 114 mg and caffeine 14.4 g; third cycle: doxorubicin 106 mg, ifosfamide 17.5 g and mesna 10.5 g. Total doses administered were cisplatin 520 mg/m^2^, doxorubicin 250 mg/m^2^ and ifosfamide 16 g/m^2^, and mesna 9.6 g/m^2^, respectively. Complications including elevation of liver enzymes, hemorrhagic cystitis, auditory disorder, and pancytopenia were observed. In cases with chemotherapy induced severe pancytopenia, the dose of doxorubicin was reduced in the subsequent cycle. 

**Figure 6:**
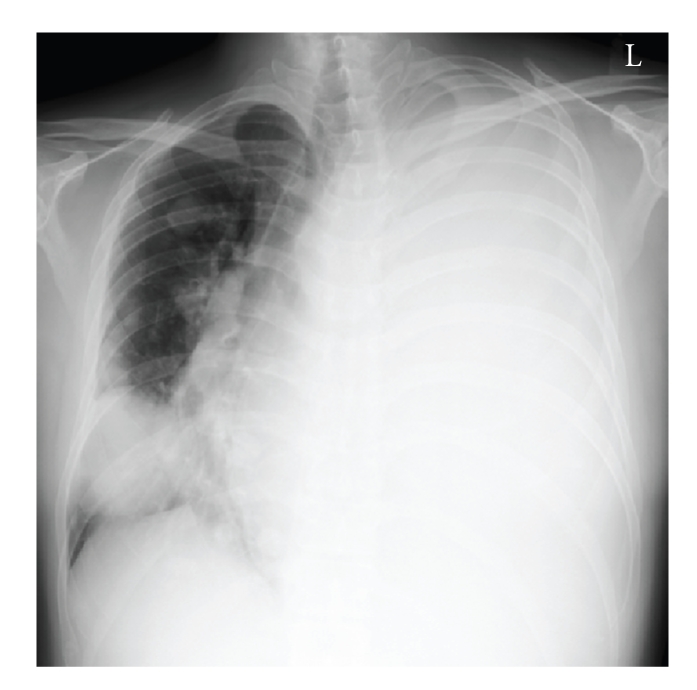
Plain radiography of lung at the final follow-up. Secondary lesion in left lung grew remarkably, and mediastinum was pressured severely.

Response to chemotherapy was evaluated after three cycles in 2 months. According to RECIST (response evaluation criteria in solid tumors), the tumor in the thigh was regarded as a stable disease (SD) and the pulmonary lesion as a progressive disease (PD). There were no indications for surgery, and the patient and his family desired more chemotherapy. Therefore, one more chemotherapy cycle (a combination of doxorubicin, ifosfamide, and mesna) was given, and the response was the same as the previous cycles. Nevertheless, the pulmonary lesion maintained its momentum conclusively occupying the left lung (Figure 6) and causing breathing difficulties. After all possible palliative care was taken, the patient died 8 months after his first visit.

### 2.4. Autopsy

**Figure 7:**
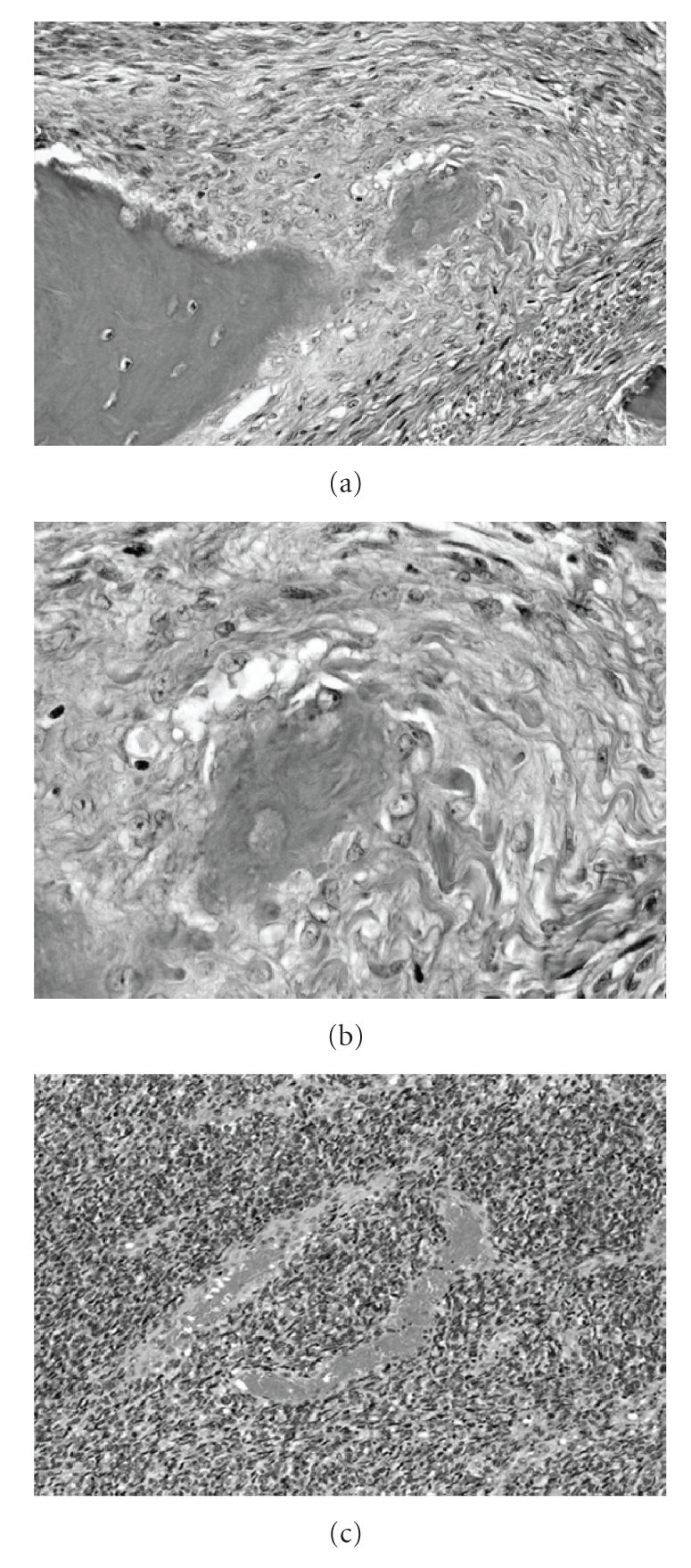
Histological section. (a) tumor of the thigh. Formation of osteoid was observed (×200). (b) strong magnification of Figure 7(a). (×400) (c) metastatic lesion (×200).

An autopsy was performed. The tumor was white and about 15 × 10 × 10 cm in diameter. The underlying bone periosteum was not involved. There were numerous pulmonary metastatic lesions, especially in the left lung. Both lungs were almost collapsed. No other metastasis was found. Histopathological sections of the thigh tumor and one of the pulmonary lesions showed proliferation of spindle-shaped tumor cells focally forming a lace-like osteoid material. The other pulmonary metastasis sites did not have any osteoid material (Figure 7). We made a definite diagnosis of ESOS.

## 3. Discussion

ESOS is a relatively rare, high-grade malignant soft tissue neoplasm. The 5-year survival rate is as low as 25% to 37% [5, 6]. It occurs mostly in soft tissue of the thigh (47% of cases) [7, 8], but can occur in any part of body [9]. It occurs predominantly in patients older than 40 years of age, whereas conventional osteosarcoma of bone usually occurs in the first or second decade of life [5–7, 10]. The patient reported here was 35 years old, slightly younger than the usual age. 

 Mechanical injury has been considered as a causative agent [11]. As with other neoplasms, the etiology is difficult to determine. ESOSs have been reported to have occurred in previously irradiated areas [12]. There was no evidence of previous trauma or irradiation in the present case. 

Microscopically, unlike in myositis ossificans where the most mature portion is located at the periphery, in ESOS, there is often a “reverse zoning phenomenon”, that is, central deposition of osteoid material and atypical spindle cell proliferation at the periphery. As with this case, reverse zoning phenomenon was observed.

 ESOS is generally a high-grade sarcoma and seems to be relatively chemoresistant, whereas osteosarcoma of bone is generally considered to be a relatively chemosensitive form of sarcoma [13]. Furthermore, Ahmad et al. suggested that both cisplatin-based chemotherapy and doxorubicin-based chemotherapy are not active against ESOS [1]. Therefore, they asserted that ESOS should be treated as therapeutically distinct from conventional osseous osteosarcoma [1]. Goldstein-Jackson et al. reported that ESOS is better treated with more aggressive multi-agent chemotherapy, including doxorubicin, ifosfamide, cisplatin and possibly methotrexate, and together with adequate surgery [14]. In the present case, however, a combination of cisplatin, doxorubicin, ifosfamide, and caffeine was not effective.

Autopsy revealed metastasis only in lungs, and no metastasis was found elsewhere. Sordillo et al. reported that distant metastasis was found in 36 of 48 patients with ESOS, and the metastasis was to the lungs in all 36 cases [15]. Therefore, the lung is the prime site to which ESOS metastasizes. In the present case, though there were secondary lesions in both lungs, osteoid material was clearly observed. 

We have reported a case of extraskeletal osteosarcoma and autopsy findings. It had already metastasized to the lungs at the first visit to our hospital and did not metastasize to any other locations. The lung tumors continue to develop despite multi-agent chemotherapy, causing death within 8 months. One of the secondary tumors contained osteoid material.
